# Simultaneous occurrence of distant metastases to the small intestine and the thoracic esophagus from anaplastic thyroid carcinoma: a case report

**DOI:** 10.1186/s40792-015-0066-9

**Published:** 2015-08-12

**Authors:** Makoto Kobayashi, Hidenori Itabashi, Tatsuru Ikeda, Norikazu Yamazaki, Tomohito Kaji, Akinori Takagane

**Affiliations:** Surgical Division, Hakodate Goryoukaku Hospital, 38-3 Goryoukaku-cho, Hakodate City, Hokkaido 040-8611 Japan; Pathological Division, Hakodate Goryoukaku Hospital, 38-3 Goryoukaku-cho, Hakodate City, Hokkaido 040-8611 Japan; Division of Otorhinolaryngology, Hakodate Goryoukaku Hospital, 38-3 Goryoukaku-cho, Hakodate City, Hokkaido 040-8611 Japan; Division of Radiology, Hakodate Goryoukaku Hospital, 38-3 Goryoukaku-cho, Hakodate City, Hokkaido 040-8611 Japan

**Keywords:** Anaplastic thyroid carcinoma, Small intestine, Esophagus, Metastases

## Abstract

Anaplastic thyroid carcinoma (ATC) is an aggressive malignancy and characterized by spreading to regional lymph nodes and distant metastases, but we were unable to find a previous report of simultaneous metastases of transformed ATC to either the small intestine or thoracic esophagus in the English language literature. A 60-year-old man suffered from well-differentiated thyroid carcinoma and underwent total thyroidectomy. Eight years later, local recurrence of thyroid cancer showed intense fluorodeoxyglucose/positron emission tomography (FDG-PET) uptake at the paratracheal region, which was suspected as a remnant tumor of the thyroid that transformed from differentiated to ATC. At that time, the patient underwent resection of the small intestine to remove an abdominal mass and consequently developed stenosis of the thoracic esophagus caused by the esophageal tumor. Histological scrutiny of specimens from both tumors in the small intestine and thoracic esophagus demonstrated the same pattern as that of undifferentiated carcinoma. Regarding histological verification and a change in the FDP-PET uptake level, it is strongly possible that our case demonstrated coincident metastases of ATC to both the small intestine and esophagus. In conclusion and to the best of our knowledge, this report is the first to present evidence suggesting that ATC has the potential to metastasize to any organs, including the digestive tract.

## Background

Anaplastic thyroid carcinoma (ATC) is an extremely aggressive human malignancy with a very poor prognosis. It is usually characterized by the spread to regional lymph nodes and distant metastases to other organs, most commonly to the lungs and bones [1]. However, metastases to the digestive tract is considered quite rare [2]. To the best of our knowledge, this case is the first report of ATC with simultaneous isolated metastases to the small intestine and thoracic esophagus, which had possibly transformed through a former instance of differentiated thyroid cancer in this patient.

## Case presentation

**Fig. 1:**
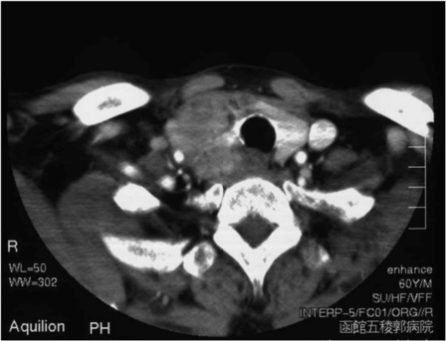
Computed tomography. Thyroid tumor with a diagnosis as a well- differentiated papillary carcinoma, invaded the cervical esophagus and right jugular vein with lymph node metastasis in July 2004

**Fig. 2:**
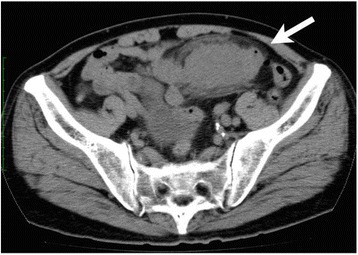
Computed tomography. Abdominal computed tomography showing an intra-abdominal abscess adjacent to the small intestine

**Fig. 3:**
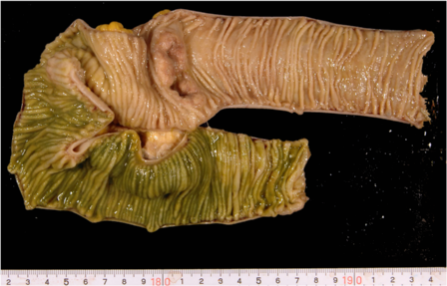
Macroscopic findings of the resected small intestine indicated a type 2 tumor, which perforated and developed a mesenteric abscess

A 60-year-old man underwent total thyroidectomy with bilateral neck dissection in July 2004 for the treatment of well-differentiated papillary thyroid carcinoma (PTC) (Fig. 1). The pathological classification was pT4a (esophagus), pN1a, M0, stage IVA. In September 2006, local recurrence to the neck (left of paratracheal sites) and distant metastasis to the femur were observed by positron emission tomography/computed tomography (PET/CT) with fluorodeoxyglucose (FDG). The patient rejected our proposed treatment regimen of resection at the local site recurrence and internal radiation therapy against the distant metastasis and was thus required to start specific substance of Maruyama (SSA). Thus, only external radiation therapy to both regions was administered as a palliative therapy. A trial of suppressive therapy with levothyroxine was also initiated. Follow-up CT in May 2007 still indicated the existence of metastatic tumor at left side of trachea without changing its size. Until December 2010, there was no evidence of growth of the neck tumor or new recurrence site observed by CT scan. In July 2012, the patient (68 years old) developed persistent abdominal pain accompanied by a fever of 38 °C. At the time of admission to our hospital, an emergent CT scan revealed an intra-abdominal mass that partially involved the adjacent small bowel (Fig. 2) and the abdominal symptoms worsened; therefore, we decided to perform emergent exploratory laparotomy. Intraoperatively, we identified an intra-abdominal abscess with a 5 cm diameter adjacent to the mesenteric vessels with partial involvement of the small intestine. The mass was completely excised together with partial resection of the small intestine. Macroscopically, the tumor was mainly composed of infectious tissue and an abscess caused by perforation of a small intestinal tumor, which extended through the muscularis propria of the small intestine and connected to the abscess cavity (Fig. 3). Histologically, the tumor was diagnosed as undifferentiated carcinoma. The postoperative course was uneventful, and the patient was discharged on postoperative day 12. Following the pathological results of the small intestinal tumor, we started to apply chemotherapy using FOLFIRI regimen [3] until March 2013.

**Fig. 4:**
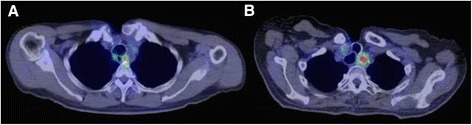
PET/CT depicting increased of FDG uptake at the paratracheal site. **a** PET/CT in 2006. **b** PET/CT in 2012

**Fig. 5:**
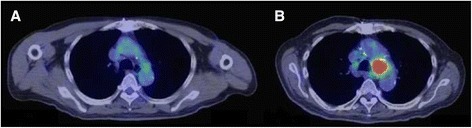
PET/CT depicting increased of FDG uptake at the intrathoracic esophagus site. **a** PET/CT in 2006 showed no accumulation of FDG around the middle mediastinum. **b** PET/CT in 2012 indicated FDG uptake on the thoracic esophagus and adjacent lymph node

During chemotherapy, a follow-up PET and CT scan in October 2012 detected progression of lymph node metastasis at the cervical paratracheal site suspected as a recurrence of the former thyroid cancer. Maximum standardized uptake value (SUVmax) of fluorodeoxyglucose-positron emission tomography (FDG-PET) at the paratracheal lesion increased from 6.0 in 2006 to 28.8 in 2012 (Fig. 4). This result suggested that the remnant PTC, which was pathologically determined as a composition of differentiated cancer cells in 2006, might have transformed to poorly differentiated or undifferentiated carcinoma. The serum thyroglobulin level and thyroid function tests (thyroid-stimulating hormone, free thyroxine) were within normal ranges. Simultaneously, FDG uptake of the thoracic esophagus at the middle mediastinum was observed positively, comparing with the find at the prior PET scan in 2006 that showed no accumulation of FDG around the thoracic esophagus (Fig. 5). Despite the deterioration of the PET results in 2012, the patient had no clinical symptoms of trouble in swallowing at that point.

**Fig. 6:**
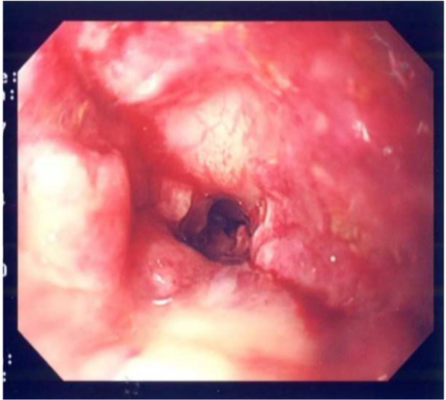
Endoscopic findings of esophageal stenosis showing a circular occlusion caused by the esophageal tumor

**Fig. 7:**
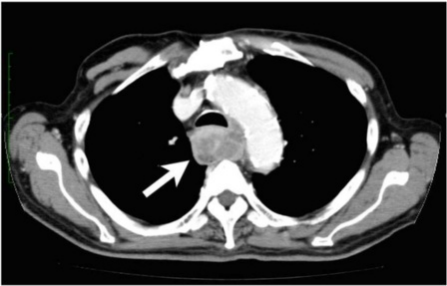
Computed tomography scan of thoracic esophageal tumor growing in the middle portion of the mediastinum at the time when the patient suffered from severe dysphagia

In March 2013, the patient developed dysphagia and was readmitted to our hospital. Endoscopic examination revealed a circular occlusion caused by an esophageal tumor (Fig. 6) at the middle part of the esophagus and a biopsy confirmed the suspicion of undifferentiated carcinoma. But we could not find any mucosal degeneration of esophageal part adjacent to the prior recurrent site of cervical parathoracic lesion. A CT scan at this time clearly indicated the esophageal tumor expanding around the middle portion of the mediastinum (Fig. 7). To relieve stenosis of the esophagus, endoscopic balloon dilation and the placement of a self-expandable metal stent were performed. Although the patient was able to swallow food, he died 9 days after endoscopic treatment due to dyspnea caused by tracheal stenosis. Permission for an autopsy was denied.

**Fig. 8:**
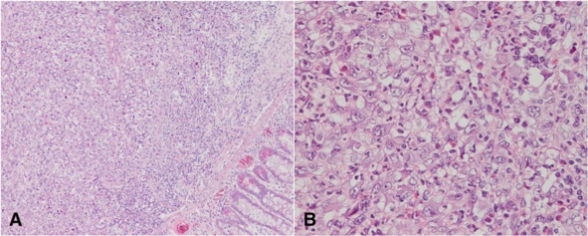
Histological findings of the tumor in the small intestine. **a** Tumor cells proliferating in the submucosal area of the small intestine (HE, ×100). **b** Anaplastic polygonal tumor cells with a prominent neutrophilic infiltrate (HE, ×400)

**Fig. 9:**
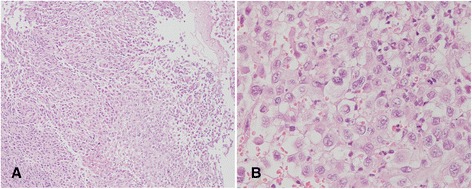
Histological findings of the biopsy specimen from the esophageal tumor. **a** Undifferentiated tumor cells with a small number of non-neoplastic squamous cells (HE, ×100). **b** Anaplastic tumor cells almost identical to those of the small intestine tumor (HE, ×400)

Histological scrutiny of both specimens from the small intestine (Fig. 8) and esophagus (Fig. 9) revealed that the tumors were similarly composed of large anaplastic polygonal cells that were discohesive and tended to dissociate from each other with a prominent neutrophilic infiltrate. There were no patterns specific of adenocarcinoma, squamous cell carcinoma, or PTC. The antibodies used in this study for immunohistochemical analysis included pankeratin (clone AE1/AE3), cytokeratin 7 (CK7), cytokeratin 20 (CK20), high molecular weight keratin (HMW-CK, clone 34Ebeta12), transcription termination factor 1 (TTF-1, clone 8G7G3/1), p53 (clone DO-7), and caudal type homeobox 2 (CDX-2, clone DAX-CDX-2), which were all obtained from DakoCytomation (Glostrup, Denmark), and thyroglobulin (polyclonal; Nichirei Biosciences, Inc., Tokyo, Japan). Cells from both tumors were strongly positive for pankeratin and p53 and negative for CK7, CK20, HMW-CK, CDX-2, TTF-1, and thyroglobulin (Table 1).

**Table 1:** Results of tumor location and immunohistochemical markers

Tumor location/markers	Pankeratin	p53	CK7	CK20	HMW-CK	CDX-2	TTF-1	Tg
Esophagus	+	+	−	−	−	−	−	−
Small intestine	+	+	−	−	−	−	−	−

*pankeratin* AE1/AE3, *CK* cytokeratin, *HMW* high molecular weight, *Tg* thyroglobulin

The pathological findings of the esophageal tumor were almost identical to those of the small intestinal tumor, which were both diagnosed as ATC. Considering the clinical progression of disease in this patient, we concluded that both tumors were derived from the same origin—a transformation of the former differentiated thyroid carcinoma treated 8 years before.

### Discussion

ATC is a rapidly growing neoplasm associated with a poor prognosis and mean survival time of 4 to 7 months [4, 5]. Approximately 20–50 % of patients with early tumor dissemination are positive for distant metastases and 90 % for adjacent tissue invasion on presentation [6]. The most common site of metastases is the lung and bone with occasional involvement of the heart, adrenals, pleura, kidneys, and pancreas [7]. A case of ATC with isolated metastasis to the small bowel is very rare, as only two cases have been reported in the PubMed database to date [8, 9]. Moreover, isolated metastasis of ATC to the thoracic esophagus is exceedingly rare, despite close proximity to the cervical esophagus. Because we found no previous reports of simultaneous isolated metastases of ATC to both the esophagus and small intestine in the literature, we presume that this is the first case report.

In some clinical situations, it is difficult to determine whether a tumor developed primarily at the site of detection or metastasized from another malignancy. Often there is no specific morphological pattern associated with an undifferentiated carcinoma; thus, such histological ambiguity makes it more difficult to identify the origin of a tumor. In the case reported in this study, microscopic findings of widely invasive growth composed of an admixture of pleomorphic giant cells and spindle cells were observed for both specimens from the small intestine and esophagus, but characteristic structures of adenocarcinoma, squamous cell carcinoma, and PTC were absent. In this case, immunohistochemical findings of positive for p53 expression and negative TTF-1 and thyroglobulin expression suggest that the tumor did not have typical patterns of either papillary or follicular thyroid carcinoma. The negative results for CK20 and CDX-2 suggested that the tumor did not likely originate from the gastrointestinal tract. Moreover, the findings of negative HMW-CK expression for both tumors indicated that these undifferentiated cells were not derived from squamous cell carcinoma of the esophagus.

Approximately 20 % of patients with ATC have a history of differentiated thyroid cancer [10], and transformation from differentiated to anaplastic cancer was previously described in a patient who was followed up with serial biopsy [11]. Moreover, mutations in the p53 tumor suppression protein associated with late de-differentiating events in an anaplastic tumor rather than a precursor from a well-differentiated tumor have been reported [12]. A recent study showed that thyroid cancer dedifferentiation is characterized by upregulation of the glucose transporter GLUT1 and a high FDG uptake [13]. Compared with other imaging modalities, PET may improve disease detection and better facilitate the management of patients with ATC [14]. The American Thyroid Association recommends FDG-PET/CT for the evaluation of metastatic disease and suggests that this modality may be useful to distinguish ATC from PTC according to the SUVmax level because of the higher FDG uptake in the former [15]. In the present case, the patient underwent treatment for advanced PTC 8 years before presentation, after which local recurrence with bone metastasis from the original PTC was identified. The latest FGD-PET showed intense uptake in a region of local recurrence of pre-existing differentiated thyroid cancer. The finding of increased activity, as indicated by a SUVmax value from 6 to 29, clearly suggests a possible transformation from differentiated thyroid cancer to ATC. The uptake of PET around the tumors of the intestine and esophagus also exhibited the same level as that of a dedifferentiated lesion.

## Conclusions

Technically, it is very difficult to confirm whether the tumor developed whether primarily or secondarily from another malignancy, if both tumors show the same pathological character. Unfortunately, we could not get permission for an autopsy and were compelled to make a final diagnosis from clinical evidence. But in conclusion, based on the histological verification and the degree of FDP uptake on PET, it is very likely that in our case, coincident metastases of ATC to both the small intestine and thoracic esophagus occurred. This case is unique because there were no previous reports on simultaneous metastases to these two organs from transformed ATC. Therefore, this report presents the first evidence suggesting that ATC has the potential to metastasize to any organs, including the digestive tract.

## Consent

Written informed consent was obtained from the patient’s family for this case report and any accompanying images. A copy of the written consent is available for review by the Editor-in-Chief of this journal.

## Abbreviations

ATC: anaplastic thyroid carcinoma
CDX-2: caudal type homeobox 2
CK: cytokeratin
FDG-PET: fluorodeoxyglucose-positron emission tomography
HMW-CK: high molecular weight cytokeratin
PTC: papillary thyroid carcinoma
TTF-1: transcription terminal factor 1
